# Epithelial Cell Proliferation Arrest Induced by Lactate and Acetate from *Lactobacillus casei* and *Bifidobacterium breve*

**DOI:** 10.1371/journal.pone.0063053

**Published:** 2013-04-30

**Authors:** Takahiro Matsuki, Thierry Pédron, Béatrice Regnault, Céline Mulet, Taeko Hara, Philippe J. Sansonetti

**Affiliations:** 1 Unité de Pathogénie Microbienne Moléculaire, Institut Pasteur, Paris, France; 2 INSERM U 786, Institut Pasteur, Paris, France; 3 Chaire de Microbiologie et Maladies Infectieuses, Collège de France, Paris, France; 4 Genopole, Institut Pasteur, Paris, France; 5 Yakult Central Institute for Microbiological Research, Tokyo, Japan; Wadsworth Center, New York State Department of Health, United States of America

## Abstract

In an attempt to identify and characterize how symbiotic bacteria of the gut microbiota affect the molecular and cellular mechanisms of epithelial homeostasis, intestinal epithelial cells were co-cultured with either *Lactobacillus* or *Bifidobacterium* as *bona fide* symbionts to examine potential gene modulations. In addition to genes involved in the innate immune response, genes encoding check-point molecules controlling the cell cycle were among the most modulated in the course of these interactions. In the m-ICcl2 murine cell line, genes encoding cyclin E1 and cyclin D1 were strongly down regulated by *L. casei* and *B. breve* respectively. Cell proliferation arrest was accordingly confirmed. Short chain fatty acids (SCFA) were the effectors of this modulation, alone or in conjunction with the acidic pH they generated. These results demonstrate that the production of SCFAs, a characteristic of these symbiotic microorganisms, is potentially an essential regulatory effector of epithelial proliferation in the gut.

## Introduction

The human intestinal tract contains a diverse community of microbes reaching up to 10^11^ bacteria/ml in the colon [1]. The intestinal microbiota serves essential functions in food digestion, metabolism of endogenous and exogenous compounds, immunomodulation, and establishment of a barrier effect that prevents colonization by pathogens. It is also involved in the regulation of intestinal homeostasis [2], impacting nutrient absorption, the quality of the physical barrier imposed to the resident microbiota by the epithelial lining, and the restitution process that requires proper balance between proliferation, differentiation and death [3]–[5]. Intestinal stem cells confined to the crypt bottom produce a progeny of epithelial cells, particularly enterocytes and goblet cells, that migrate upwards along the villus axis in the small intestine and to the epithelial surface in the colon. These cells initially constitute a proliferative compartment, but as migration progresses upwards, cell division arrests, final differentiation is completed, and cells eventually undergo apoptosis before sloughing off into the lumen. The epithelium is exposed to the luminal microbiota, thereby offering opportunities for bacteria or bacterial products to affect the dynamics of the crypt-to-surface axis and to play a role in epithelial restitution.

Mono-contamination of germ-free animals (i.e. gnotobiotic) has been pivotal in elucidating the contribution of the gut microbiota to gut epithelial homeostasis. Early studies demonstrated a number of morphological differences in the histological aspect of the intestinal tract of germ-free (GF) versus gnotobiotic or conventional (CV) mice. CV mice display regular and uniform villi, whereas GF mice display irregular villi. This is well in line with early studies showing that the presence of an intestinal flora provided mice with a two-fold increase in rate of epithelial turn over [6]. In addition, GF animals show a thinner lamina propria, a slower epithelial turnover, slender villi, and a lower activity of digestive enzymes than CV mice [7]–[9]. Analysis of the bacterial effectors and signaling pathways that affect epithelial homeostasis has begun [10], and a cellular microbiology of symbiosis is on its way [11]. In order to examine how luminal bacteria affect gut epithelial proliferation, differentiation and death, we established an *in vitro* assay in which intestinal epithelial cells were exposed to *Lactobacillus casei* or *Bifidobacterium breve* used as *bona fide* symbionts. Our previous experiments have shown that in a model of human Caco-2 cells, *L. casei* strongly down-regulated the pro-inflammatory signals induced by an invasive strain of *Shigella* [12]. Beyond the issue of innate immune regulation, we wanted here to address the molecular basis of epithelial homeostasis in the presence of bacterial symbionts.

This work shows that *Lactobacillus* and *Bifidobacterium* modulate cell cycle gene expression in human and murine epithelial cell lines and that short chain fatty acids (SCFA) represent major effectors of this modulation, alone or via the acidic pH they generate.

## Results

### Gene Expression Modulation of Caco-2 Cells by *L. casei* and *B. breve*

**Figure 1:**
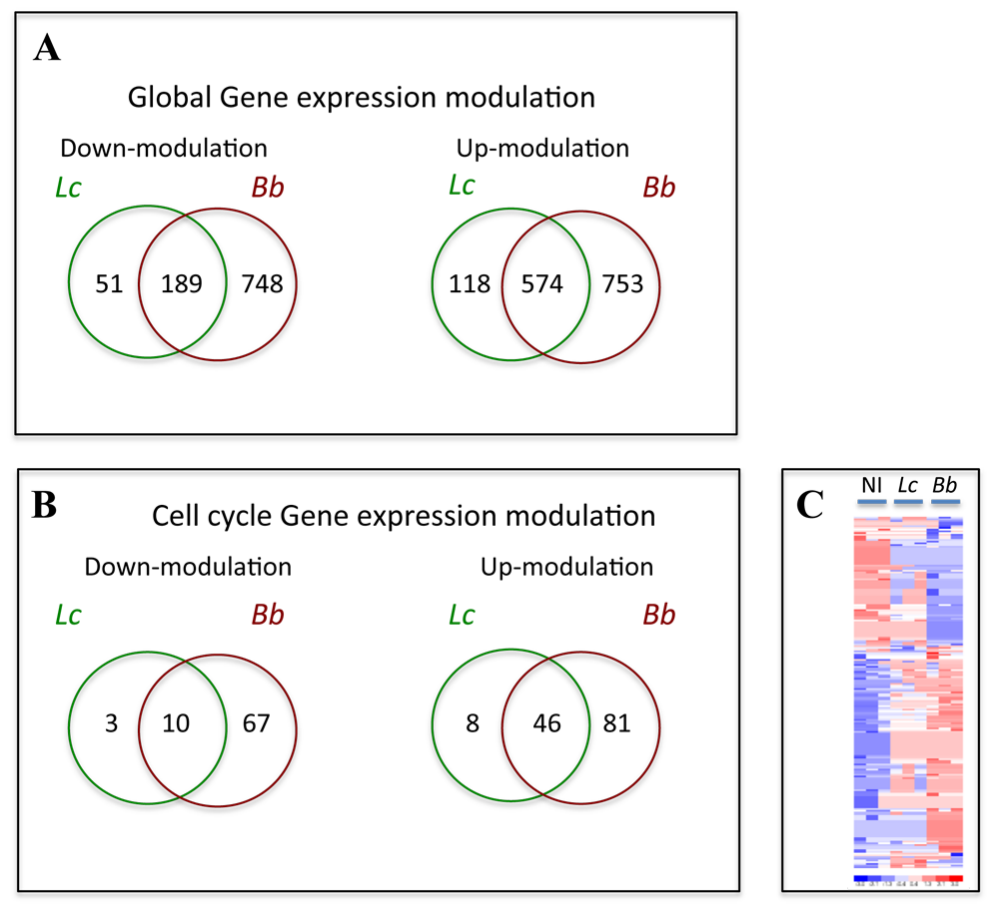
Caco-2 cells gene expression. After co-culture of Caco-2 cells with *L casei* and *B. breve* and hybridization on Human U133A genechip, results were normalized with RAM and analysis was performed using dChip software as described in material and method section. Significantly modulated genes (fold change >1,75 and p-value <0,05) are shown. Numbers indicate the modulated genes. A: global gene expression modulation; B: cell cycle gene expression modulation; C: Hierarchical clustering of cell cycle gene expression was performed using dChip software with Euclidian distance and average as a linkage method. Before clustering, expression values for one gene across all samples were standardized to produce a mean of zero. Increased or decreased values were then compared with that mean. Red and blue colors represent expression that is higher or lower than the mean value, respectively. The key for intensity of expression is indicated under the bar. See Table S2 for the corresponding fold change and p-value of cell cycle modulated genes.

To analyze gene expression in IEC exposed to symbiotic bacteria, human intestinal Caco-2 cells were co-cultured overnight with the *L. casei* strain DN-114 001 and the *B. breve* strain DN-156 007 at a multiplicity of infection (MOI) of 100. Transcriptional profiling performed with the Affymetrix GeneChip technology showed the down-regulation of 988 genes and the up-regulation of 1445 genes by a factor of 1.75 or more, as shown in Fig. 1A and listed in Table S1. Interestingly, using Gene Ontology definition, among the different signaling and metabolic pathways modulated by these bacteria, 80 and 135 genes encoding key factors of the cell cycle were respectively down- or up-regulated, including cyclin D1, cyclin E1, growth arrest and DNA damage, cullin 1 (Fig. 1B–C, and Table S2). *B. breve* induced stronger modulation of gene expression than *L. casei*, however many genes were modulated by the 2 strains such as MNAT1 which is involved in the CDK-activating kinase complex with cyclin H and CDK7. MNAT1 and CDK7 had fold change of 13 and 5 respectively. Genes involved in controlling the G1/S transition of the mitotic cell cycle, such as cyclin D1, cyclin E1 and cullin 1 were up-regulated by *B. breve*, p57 (cyclin-dependent kinase inhibitor 1C, Cdkn1c, Kip2) was down-regulated by the two strains with a fold-change of 3. These *in vitro* data indicated that *L. casei* and *B. breve* had the capacity to affect the epithelial proliferative compartment thus significantly impacting epithelial homeostasis.

### *L. casei* Down-regulates Cyclin E1 While *B. breve* Down-regulates Cyclin D1 and Cyclin E1 Gene Expression *in vitro*

**Figure 2:**
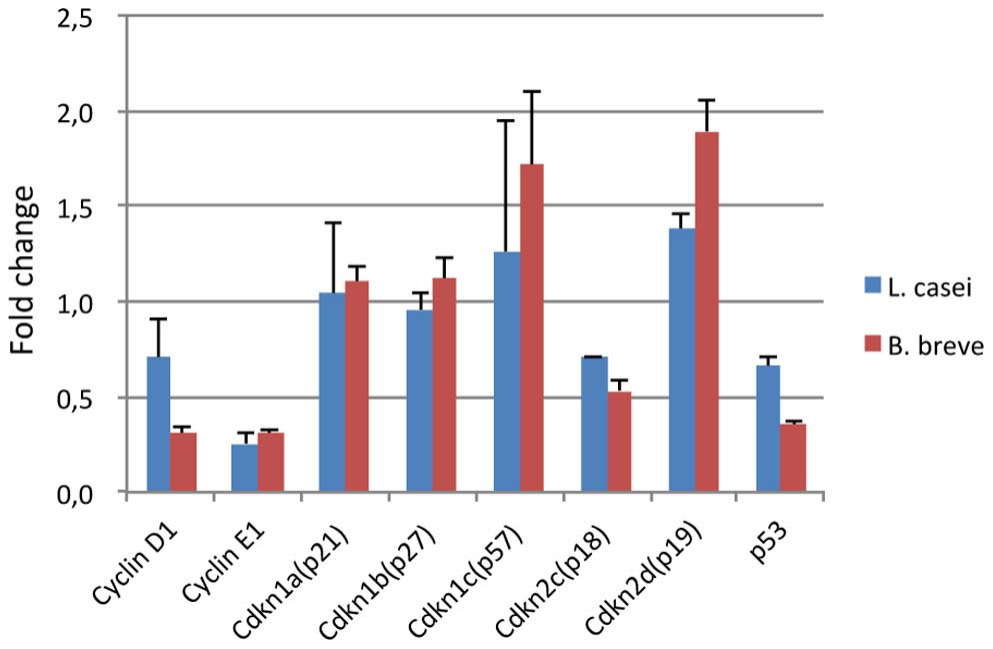
Fold change of gene expression after overnight co-culture of m-ICcl2 with *L.casei* and *B.breve*. qRT-PCR was analyzed by the ddCt method using m-ICcl2 alone and GAPDH as reference.

To elucidate the mechanisms by which bacterial symbionts affect cell cycle-related gene expression in the epithelium, we switched from the Caco-2 human cancer cell line which has mutations in cell cycle check point systems [13], to the non-cancerous transformed murine intestinal crypt cell line m-ICcl2 [14]. m-ICcl2 cells were co-cultured at 40 to 50% confluence with the *L. casei* strain Shirota and the *B. breve* strain Yakult at a MOI of 100. Real-time PCR with cell cycle-related gene targeted primers was performed and the results are shown in Fig. 2. Interestingly, the responses of m-ICcl2 cells differed between *L. casei* and *B. breve*: *L. casei* down-regulated the expression of the cyclin E1 gene, whereas *B. breve* down-regulated the expression of cyclin D1 and cyclin E1 genes, both of which playing a key role in the regulation of the G1/S check point. When time course experiments were performed (2, 4, 8, 16 hours), slight gene expression modulation began to appear after 8 hours. In contrast, the level of gene expression of Cdkn1a (p21), Cdkn1b (p27) and Cdkn2c (p18) remained unchanged. Expression of the p53 gene was reduced to 0.73±0.12, in co-culture experiments with *L. casei*, and to 0.35±0.02 in co-cultures with *B. breve*. These two bacterial strains induced a slight up-regulation of expression of Cdkn2d (p19) and Cdkn1c (p57) which encode two major cyclin-dependent kinase inhibitors. Interestingly the type strain of *L. casei* (CIP 107868, ATCC 334) also induces the same gene expression modulation, indicating a common feature among different isolates of this lactic acid bacteria (data not shown).

### *L. casei* and *B. breve* Induce Increased Expression of Differentiation Genes in m-ICcl2 Cells

*L. casei* and *B. breve* not only down-regulated cell cycle gene expression, but also induced an increased in expression of genes encoding proteins that characterize a state of higher IEC differentiation. Among these genes, two were particularly induced: the gene encoding the poly-Ig receptor, involved in the uptake and transport of dimeric IgAs [15]–[17] was up-regulated by a factor of 2,48±0,40 and 2,01±0,47 by *L. casei* and *B. breve* respectively. and the gene encoding intestinal alkaline phosphatase, another key differentiation marker of maturation of the digestive capacities of the brush border, up-regulated during IEC differentiation [18], was increased by 3,63±0,44 and 2,67±0,23 following co-culture of m-ICcl2 with *L. casei* and *B. breve* respectively.

### Soluble Factors are the Causative Effectors of Changes in Cell Cycle-related Gene Expression

**Figure 3:**
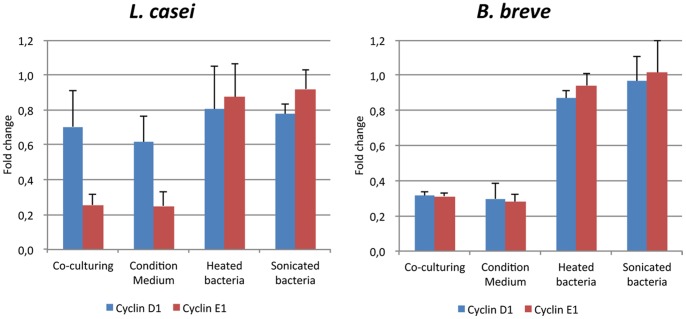
Identification of effectors that affect m-ICcl2 cell cycle related gene. qRT-PCR was analyzed by the ddCt method using m-ICcl2 alone and GAPDH as reference.

In order to identify the bacterial effectors that modulate the expression of cell cycle related genes, conditioned medium, heat-treated bacteria, and sonicated bacteria were prepared as described in the experimental procedures. *L. casei*- and *B. breve*-conditioned media showed effects similar to living bacteria, whereas heat-treated or sonicated bacteria showed no effect on the transcription of the cyclin genes (Fig. 3). These observations indicate that the cells were affected by media conditions and/or soluble bacterial factors and that Microbial-Associated Molecular Patterns (MAMPs) and bacteria-cell contact did not account for the down-regulation of cell cycle genes expression. Additional experiments with heat-treated CM and amicon filtrated CM used as inducers indicated that the effectors were heat-stable, and their molecular weight was less than 3 kDa (data not shown).

### Identification of Acetate and Lactate as Regulators of Cyclin D1 and Cyclin E1 Gene Expression

**Figure 4:**
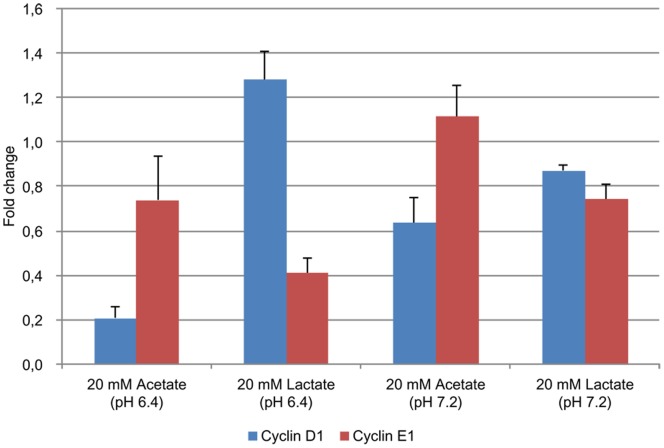
Impact of acetate and lactate on m-ICcl2 cyclin D1 and cyclin E1 gene expression. qRT-PCR was analyzed by the dδCt method using m-ICcl2 alone and GAPDH as reference.

According to these results, we hypothesized that lactate and/or acetate produced as a result of fermentation by *Lactobacillus* and/or *Bifidobacterium* could be the relevant effectors. Indeed, HPLC analysis showed that 18 mM lactate was detected in the *L. casei*-conditioned medium, while 14 mM acetate, 6 mM lactate, and 1 mM formate were measured in *B. breve*-conditioned medium. Butyrate and propionate were not detected in these conditioned media. The effects of lactate and acetate on cyclin D1 and cyclin E1 gene expression were evaluated through the addition of acetate, lactate, sodium acetate, or sodium lactate to the culture medium. As hypothesized, down-regulation of cyclin E1 was observed in the presence of lactate, whereas cyclin D1 was down-regulated by acetate at pH 6.4 (Fig. 4). We then evaluate the effects of pH by adding sodium acetate and sodium lactate to the culture medium. As a result, the down-regulation of cyclin E1 gene expression by lactate was not observed at pH 7.2, indicating that it was induced by acidic pH rather than by the lactate molecule itself. Down-regulation of cyclin D1 by acetate was still observed at pH 7.2, but the effect was not as marked as that obtained at a lower pH.

As the monocarboxylate transporter MCT1 transports lactate into cells [19], we inhibited its expression by siRNA transfection in order to see if silencing could reverse the down-regulation of cyclin E1 gene expression induced by *L. casei*-produced lactate. Even with a down-regulation of MCT1 gene expression in m-ICcl2 cells with a fold change of 5,89 after MCT siRNA transfection versus a fold change of 1,08 after control siRNA transfection, the down-regulation of cyclin E1 induced by *L. casei* was still observed (with a equal fold change of 11 after transfection with MCT1 siRNA or control siRNA), indicating that silencing this main lactate transporter was not enough to block the effect of *L. casei* on cyclin E1, thus confirming the primary role of the acidic pH in the regulatory process.

### Acetate and Lactate Induce Cell Proliferation Arrest in a Concentration- and pH-dependent Manner

**Figure 5:**
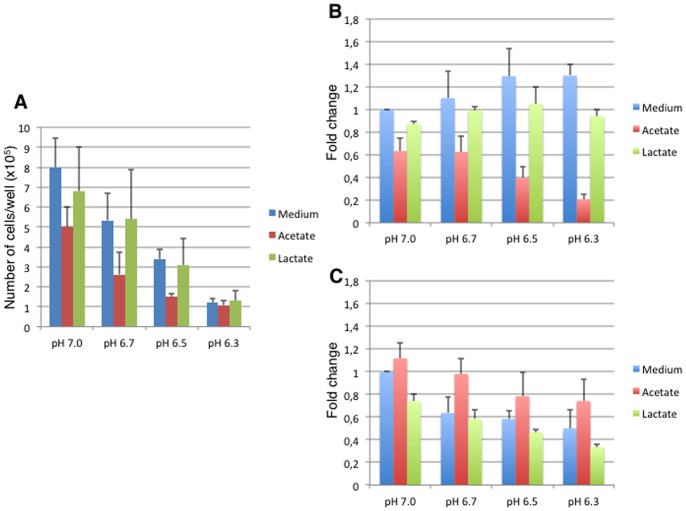
Acetate induces cell proliferation arrest in a concentration & pH dependent manner. A: Number of m-ICcl2 per well after incubation at different pH with 20 mM of SCFA. Number represent cell counts (x10^5^) per well. B-C: qRT-PCR of cyclin D1 (B) and cyclin E1 (C) gene expression after incubation of m-ICcl2 with or without 20 mM acetate or lactate at different pHs. qRT-PCR was analyzed by the dδCt method using m-ICcl2 alone and GAPDH as reference.

To decipher whether or not the down-modulation of cyclins gene expression was accompanied by cell growth arrest, m-ICcl2 cells were seeded at 10^5^ per well and incubated for 3 days at various pH, with or without 20 mM acetate or lactate added to the medium. As shown in Fig. 5A, though the growth of m-ICcl2 cells was similarly arrested by the decrease of the pH or by the presence of lactate, acetate induced a cell growth arrest significantly different than that caused by pH changes alone, at 20 mM. Indeed from pH 7 to pH 6.5 we observed a proliferation decrease of 40% in the presence of 20 mM acetate. The effect of acetate was clearly concentration dependent as 5.10^5^ cells were counted at pH 7 in wells containing 20 mM acetate, and 7.10^5^ and 8.10^5^ cells were counted in wells containing cells with or without 10 mM acetate. This result correlated with the occurrence of a decreased level of cyclin D1 gene expression, which was not down-regulated by acidic pH alone, but was down-regulated in a pH-dependent manner in 20 mM acetate (Fig. 5B). Whereas for cyclin E1 the down-regulation observed was mainly due to a pH effect following acidification of the medium by the bacteria (Fig. 5C).

**Figure 6:**
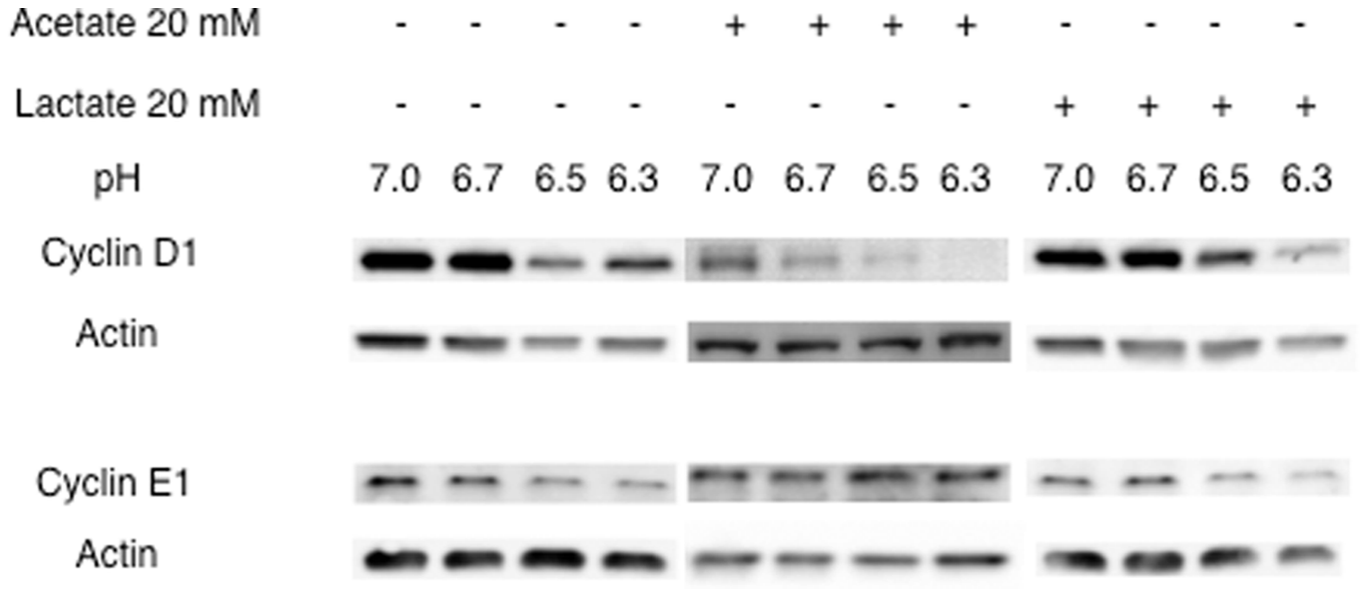
Acetate and pH induces respectively a down-regulation of cyclin D1 and cyclin E1 proteins. Western blots were performed after 16 hr co-culture of m-ICcl2 cells with 20 mM of acetate and lactate at different pHs. For the detection of cyclin E1, cells were synchronized by a double thymidine block treatment prior co-culture as described in material and methods section.

Analysis of cyclin D1 and E1 expression at the protein level by Western blot clearly validated the former results. Cyclin D1 was slightly diminished at pH 6.3, acetate clearly down-regulated its level below pH 6.7, whereas the level of cyclin E1 decreased at lower pH (Fig. 6).

## Discussion

Regulation of the cell cycle is a growing theme in microbial pathogenesis, but has yet to be significantly addressed in the context of symbiotic relationships. In microbial pathogenesis many bacterial effectors called cyclomodulins were described as modulators of the eukaryotic cell cycle [20], [21]. *Bacillus anthracis* and *Bordetella pertussis* secrete adenylate cyclase toxins and *Escherichia coli* produce subtilase cytotoxin that induce arrest of macrophage proliferation by inducing a reduction of the amount of cyclin D1 [22], [23]. Colibactin and cycle inhibiting factor (Cif) produced by enteropathogenic *E. coli* induce a cell-cycle arrest [24]–[26]. The IpaB effector secreted by *Shigella* inhibits mitosis [27]. Interestingly, two effectors secreted by *Helicobacter pylori* induce opposite effect on epithelial gastric cells. Whereas the vacuolating cytotoxin (VacA) inhibits cell proliferation through a p53-dependent pathway [28], the cytotoxin-associated gene A (CagA) protein increases cyclin D1 expression, thereby inducing cell progression from the G1 to S phase [29]. Bifidobacteria and lactic acid bacteria including *L. casei*, can be used as *bona fide* models of symbionts to study how the microbiota affects the homeostasis of the intestinal epithelium. These bacteria also fall within the category of ‘probiotics’, due to certain immunomodulatory properties, and to a capacity to protect against certain infectious and inflammatory conditions of the gut. Some data also suggest a protective effect against oncogenesis [30], [31]. Following our initial demonstration that *L. casei* was able to protect against the potent pro-inflammatory properties of *S. flexneri* [12], we decided to pursue an unbiased study of the effect of *L. casei* and *B. breve in vitro* on IEC by analyzing the alteration in gene expression profiles observed upon co-culture of Caco-2 cells with these two species, using GeneChip technology. Unexpectedly, analysis of the data indicated that key effectors of the cell cycle like cyclin D1, cyclin E1, growth arrest and DNA damage, and cullin 1, were the primary targets of the transcriptional modifications imposed on these tumor cells.

Based upon this preliminary evidence which indicates that the cell cycle may be modulated by symbiotic microorganisms, we decided to confirm the observation in a transformed but non tumor cell line, and to identify the relevant bacterial effector (s).

We selected the murine small intestinal crypt cell line m-ICcl2 which is well adapted to study microbial cell interactions [16], [32] allowing future experimental work *in vivo*. Hence m-ICcl2 cells were co-cultured with *L. casei* and *B. breve*. Real-time PCR with primers targeted to cell cycle genes showed that *L. casei* also down-regulated gene expression of cyclin E1, whereas *B. breve* down-regulated cyclin D1 and E1 gene expression, both cyclins being essential as G1/S check points. We then identified acetate and/or lactate as potential primary effectors inducing transcriptional repression of the cyclin D1 and cyclin E1 genes. These data were confirmed at the protein expression level.

Fermentation metabolites of the intestinal microbiota such as SCFAs play an important role in the gut physiology [33], [34]. Acetate, propionate and butyrate as fermentation end products are assimilated by the mammalian host. The concentration and composition of SCFAs vary among individuals. SCFA concentration in the lumen is in the range of 70–130 mM [35], with molar ratios of acetate:propionate:butyrate varying from approximately 75∶15∶10 to 40∶40∶20 [36]. It has been estimated that SCFAs can contribute to about 10% of the total caloric requirements in humans [37]. In addition to its role as a fuel, butyrate is notable for its function as an inhibitor of histone deacetylases, leading to hyperacetylation of chromatin, thereby influencing gene expression [38]. Concerning the effect of lactate, when the SCFA transporter MCT-1 was silenced using siRNA, no change in the down-regulation of cyclin E1 by *L. casei* was observed, indicating that the observation in this study was not due to the transport and possible use of lactate as dominant carbon source in cells exposed to *L. casei* or *B. breve*. The effect of lactate and acetate on gene expression in IEC has not been reported in comparison to butyrate on epithelial growth and differentiation [39]. More recently, Fukuda et al [40] showed that a luminal increase in acetate concentration induces a protective effect against a lethal infection by *E. coli* O157 through the inhibition of the translocation of Shiga toxin. The mechanism remains to be characterized.

Although further investigation is needed to clarify the detailed molecular pathways involved in cell cycle control by *L. casei* and *B. breve*, our results indicate that the effects of symbiotic bacteria on the gut epithelium vary among the species of symbionts studied and that the nature and balance of the organic acids they produce may play a dominant role by variably regulating epithelial proliferation. Our results also reveal that the biological effect of these organic acids is largely mediated by the acid environment they provide to exposed cells. In our attempt to identify the molecular mediators of the symbiotic interactions established between the microbiota and the epithelium, the cellular response to acid pH stress thus turns out to be a key element of the cross talk with lactobacillaceae. How cells cope and regulate acidity is a well-studied theme, particularly in the intestinal epithelium and the renal tubular epithelium [41]. On the other hand, little is currently available in the literature on how cells respond at the transcriptional, translational and post-translational level to prolonged exposure to low pH [42]. Cell cycle arrest appears to be a dominant element in the cellular response, as a possible mean to minimize the extent of possible alterations imposed on a proliferating cell population. More fundamental work is needed to address this issue and its *in vivo* relevance and consequences needs further analysis.

The SCFA-induced expression of cell cycle regulators such as p19, p57, and the transcriptional factor GATA-2 that represses the expression of cyclins, including cyclin D1 and also the concomitant induction of cell differentiation indicate that these symbionts impose a complex interplay with the epithelium where intricate mechanisms controlling the cell cycle and mechanisms stimulating cell differentiation. These results are the basis for future *in vivo* studies which shall confirm the extent to which colonization of the gut mucosa by *L. casei* and *B. breve* affect the homeostasis of the epithelium.

## Materials and Methods

### Preparation of Epithelial Cell

The human intestinal epithelial cell line (IEC) Caco-2 that is derived from a colonic carcinoma was used in this study [43]. Cells were grown in an incubator at 37°C, 10% CO_2_, in Dulbecco’s modified Eagle’s medium supplemented with 10% inactivated fetal calf serum (Life Technologies), 1% non-essential amino acid, and antibiotics (penicillin-streptomycin, respectively, 100 units/ml and 100 µg/ml). Before co-culture with bacteria, cells were washed in Dulbecco’s modified Eagle’s medium without serum and incubated without antibiotics at 37°C for 2 h in the same medium.

The murine IEC line m-ICcl2 [14] was maintained at 37°C in a 5% CO_2_/95% air atmosphere in HAMF’12/DMEM (Gibco, NY, USA, v/v) containing the following reagents (Sigma): insulin (5 µg/ml), dexamethasone (5×10^−8^ M), selenium (60 nM), transferring (5 µg/ml), triiodothyronine (10^−9^M), EGF (10 ng/ml), hepes 20 mM, glutamine 2 mM, D-glucose (0.22%) and inactivated fetal calf serum (2%). 6 well plates were pre-treated with rat tail collagen (100 µg/ml) as described elsewhere [16]. Cells were seeded at 1.0×10^5^ cells/well in 2 ml of medium on a 6-well plate.

After overnight-incubation of m-ICcl2 with bacteria, cells were recovered from wells with trypsin, centrifuged and counted using a Malassez chamber. Occasionally, m-ICcl2 cells were synchronized by a double thymidine block treatment (final concentration of 25 mM), released into fresh medium, and treated with test medium for 16 hr after released.

### Bacterial Culture, Co-culture Experiments, and Conditioned Cell Culture Media (CM) Preparation

*Lactobacillus casei* strain Shirota or DN-114 001 and *Bifidobacterium breve* strain Yakult or DN-156 007 were cultured at 37°C in MRS broth (Difco, Detroit, USA) and GAM broth (Nikken Seiyaku, Tokyo, Japan), respectively. Bacteria in stationary growth phase were harvested by centrifugation (5 min at 12,000 rpm), washed twice with PBS buffer (pH 7.2), and resuspended in the growth medium of m-ICcl2 cells. Two days after seeding, cells were co-cultured at different time-point with 2.10^7^ *L. casei* or *B. breve* (Multiplicity Of Infection: 100). Results indicate 16 hr co-culture experiments. Following the incubation, culture supernatants were collected, and pH and bacterial concentration were measured. To identify specific effectors, the bacteria were incubated in m-ICcl2 medium for 16 h, bacterial pellets were collected, either sonicated or heat treated, resuspended in medium, and used as the bacterial components. Medium supernatants were filtered (pore size; 0.22 µm) and used as conditioned medium (CM). For the determination of short-chain fatty acids (SCFA), 270 µl of the medium was mixed with 30 µl of 10% HClO_4_ and incubated at 4°C overnight. The samples were centrifuged for 5 minutes at 14,000 g, filtrated with Centri-Cut (Kurabo, Osaka, Japan), and subjected to HPLC system (Waters, USA) with Rspak KC-811 column (Showa Denko K.K., Tokyo) equipped with 432 electro-conductivity detector (Waters, USA) as previously described [44].

### GeneChip Hybridization and Statistical Analysis

Caco-2 cells were incubated overnight with or without bacteria at a MOI of 100. Three biological replicates were performed for each experimental condition. Following washing in cold phosphate-buffered saline, cells were lysed, and the total RNA was extracted by RNeasy Mini kit (Qiagen, Valencia, CA). Integrity and purity of RNA was checked by spectrophotometry and capillary electrophoresis using the Bioanalyzer 2100 and RNA 6000 LabChip kit from Agilent Technologies (Palo Alto, CA). cDNA was synthesized using Superscript Choice system (Invitrogen). Biotin-labeled-cRNA was then synthesized with the Enzo BioArray High Yield RNA transcript labeling kit (Enzo Biochem, New York, NY). After purification with Rneasy columns (Qiagen), 12.5 µg of fragmented cRNA was hybridized to an HG-U133A array (Affymetrix), and the chips were automatically washed and stained with streptavidin-phycoerythrin using a fluidics station. Finally, the arrays were scanned at 570 nm with a resolution of 3 µm/pixel, using a GeneArray scanner from Agilent Technologies. Preprocessing by Robust Multichip Average (RMA) was applied to process individual probe values (perfect match) and to generate summary values for each probe set (transcript) [45]. The dChip software was used for comparative analysis and for hierarchical clustering with Euclidean distance and average as a linkage method [46]. Before clustering, expression values for one gene across all samples were standardized to produce a mean of zero. Increased or decreased values were then ranged compared with that mean. For the analysis step, redundant probe set were removed. Data have been deposited in NCBI’s Gene Expression Omnibus and are accessible through GEO Series accession number GSE37369.

### RNAs Isolation and Quantitative Real-time PCR

Total RNA was extracted from m-ICcl2 cells by using RNeasy Mini Kit (Qiagen) according to the manufacture’s instruction. A 2-µg aliquot of total RNA was reverse-transcribed using Oligo (dT) 15 Primer (Promega), RNASIN (Promega), and Superscript II (Invitrogen, Carlsbad, CA, USA). Primers used for qRT-PCR are described in Table S3. qRT-PCRs were carried out in a 15 µl volume containing 6 µl of cDNA (diluted at 1/100), specific primers (0.2 µM each), and 7.5 µl of Power SYBR Green mix (Applied Biosystems). The thermal cycling conditions were 10 min at 95°C, followed by 40 cycles of 15 sec at 95°C and 1 min at 60°C, using Applied Biosystems 7900HT. All reactions were performed in duplicate. Relative quantification of gene expression was performed using the comparative Ct method [47]. Results were normalized using the glyceraldehyde 3-phosphate dehydrogenase (GAPDH) housekeeping gene.

### MCT1 Silencing

m-ICcl2 cells were transfected with MCT1 siRNA (sc-40114, Santa Cruz), a pool of 3 target-specific 20–25 nt siRNAs or with control siRNA-A (sc-37007, Santa Cruz) following manufacturer’s instruction using siRNA transfection reagent (sc-29528, Santa Cruz). The day after transfection, cells were co-cultured o/n with or without *L. casei* at a MOI of 100, and after RNA extraction, qRT-PCR was done with MCT-1 and Cyclin E1 primers.

### Western Blot Analysis

Treated cells were lysed by the addition of 200 µl of Laemmli solution [48]. After heating for 5 min at 90°C, 10 µl of lysate was loaded in a 10% acrylamide SDS-PAGE. After migration, proteins were transferred onto Hybond N^+^ (Amersham) by semidry blotting method. After blocking with 5% milk in PBS, the membrane was incubated overnight with anti-cyclin D1/bcl-1 Ab-3 (Thermo, 1/500), cyclin E1 M-20 (Santa Cruz, 1/500), or anti-actin (Sigma-Aldrich, 1/1000) in PBS. Membranes were washed in PBS/Tween 0,1% and incubated with a peroxidase-labeled secondary antibody (1/1000) for 1 h. After washing, membranes were incubated for 5 min with ECL chemiluminescence reagent (Amersham Biosciences). Acquisitions were performed with a Molecular Imager ChemiDoc XRS System (Bio-Rad) or LAS 3000 (Fujifilm).

## Supporting Information

Table S1 Human intestinal Caco-2 cells transcriptional profiling with the Affymetrix GeneChip technology. SLR value indicates the difference in Log_2_ between the signals of Caco-2 cultivated with bacteria (*L. casei* or *B. breve*) and Caco-2 cells alone and their associated p-values.
(XLS)

Table S2 Genes encoding key factors of the cell cycle were affected by *L. casei* and *B. breve* co-culturing. SLR value indicates the difference in Log_2_ between the signals of Caco-2 cultivated with bacteria (*L. casei* or *B. breve*) and Caco-2 cells alone and their associated p-values.
(XLS)

Table S3 Primers used for qRT-PCR in this study.
(DOCX)

## References

[1] Costello EK, Lauber CL, Hamady M, Fierer N, Gordon JI, et al (2009) Bacterial community variation in human body habitats across space and time. Science 326: 1694–1697. 19892944 10.1126/science.1177486 PMC3602444

[2] Pédron T, Sansonetti PJ (2008) Commensals, bacterial pathogens and intestinal inflammation: an intriguing ménage à trois. Cell Host Microbe 3: 344–347. 18541210 10.1016/j.chom.2008.05.010

[3] Sekirov I, Russell SL, Antunes LC, Finlay BB (2010) Gut microbiota in health and disease. Physiol Rev 90: 859–904. 20664075 10.1152/physrev.00045.2009

[4] Kau AL, Ahern PP, Griffin NW, Goodman AL, Gordon JI (2011) Human nutrition, the gut microbiome and the immune system. Nature 474: 327–336. 21677749 10.1038/nature10213 PMC3298082

[5] Keeney KM, Finlay BB (2011) Enteric pathogen exploitation of the microbiota-generated nutrient environment of the gut. Curr Opin Microbiol 14: 92–98. 21215681 10.1016/j.mib.2010.12.012 PMC3039043

[6] Savage DC, Siegel JE, Snellen JE, Whitt DD (1981) Transit time of epithelial cells in the small intestines of germfree mice and ex-germfree mice associated with indigenous microorganisms. Appl Environ Microbiol 42: 996–1001. 7198427 10.1128/aem.42.6.996-1001.1981 PMC244145

[7] Hooper LV, Wong MH, Thelin A, Hansson L, Falk PG, et al (2001) Molecular analysis of commensal host-microbial relationships in the intestine. Science 291: 881–884. 11157169 10.1126/science.291.5505.881

[8] Faith JJ, Rey FE, O’Donnell D, Karlsson M, McNulty NP, et al (2010) Creating and characterizing communities of human gut microbes in gnotobiotic mice. ISME J 4(9): 1094–1098. 20664551 10.1038/ismej.2010.110 PMC2927777

[9] Tlaskalová-Hogenová H, Stěpánková R, Kozáková H, Hudcovic T, Vannucci L, et al (2011) The role of gut microbiota (commensal bacteria) and the mucosal barrier in the pathogenesis of inflammatory and autoimmune diseases and cancer: contribution of germ-free and gnotobiotic animal models of human diseases. Cell Mol Immunol 8: 110–120. 21278760 10.1038/cmi.2010.67 PMC4003137

[10] Rakoff-Nahoum S, Medzhitov R (2008) Innate immune recognition of the indigenous microbial flora. Mucosal Immunol 1 Suppl 1S10–S14. 19079220 10.1038/mi.2008.49

[11] Ohnmacht C, Marques R, Presley L, Sawa S, Lochner M, et al (2011) Intestinal microbiota, evolution of the immune system and the bad reputation of pro-inflammatory immunity. Cell Microbiol 13: 653–659. 21338464 10.1111/j.1462-5822.2011.01577.x

[12] Tien MT, Girardin SE, Regnault B, Le Bourhis L, Dillies MA, et al (2006) Anti-inflammatory effect of *Lactobacillus casei* on Shigella-infected human intestinal epithelial cells. J Immunol 176: 1228–1237. 16394013 10.4049/jimmunol.176.2.1228

[13] Gartel AL, Feliciano C, Tyner AL (2003) A new method for determining the status of p53 in tumor cell lines of different origin. Oncol Res 13: 405–408. 12725531 10.3727/096504003108748429

[14] Bens M, Bogdanova A, Cluzeaud F, Miquerol L, Kerneis S, et al (1996) Transimmortalized mouse intestinal cells (m-IC_cl2_) that maintain a crypt phenotype. Am J Physiol 270: C1666–C1674. 8764149 10.1152/ajpcell.1996.270.6.C1666

[15] Kaetzel CS, Robinson JK, Chintalacharuvu KR, Vaerman JP, Lamm ME (1991) The polymeric immunoglobulin receptor (secretory component) mediates transport of immune complexes across epithelial cells: a local defense function for IgA. Proc Natl Acad Sci U S A 88: 8796–8800. 1924341 10.1073/pnas.88.19.8796 PMC52597

[16] Fernandez MI, Pédron T, Tournebize R, Olivo-Marin JC, Sansonetti PJ, et al (2003) Anti-inflammatory role for intracellular dimeric immunoglobulin a by neutralization of lipopolysaccharide in epithelial cells. Immunity 18: 739–749. 12818156 10.1016/s1074-7613(03)00122-5

[17] Bruno ME, Kaetzel CS (2005) Long-term exposure of the HT-29 human intestinal epithelial cell line to TNF causes sustained up-regulation of the polymeric Ig receptor and proinflammatory genes through transcriptional and posttranscriptional mechanisms. J Immunol 174: 7278–7284. 15905574 10.4049/jimmunol.174.11.7278

[18] Olsen L, Bressendorff S, Troelsen JT, Olsen J (2005) Differentiation-dependent activation of the human intestinal alkaline phosphatase promoter by HNF-4 in intestinal cells. Am J Physiol Gastrointest Liver Physiol 289: G220–G226. 15831710 10.1152/ajpgi.00449.2004

[19] Boidot R, Végran F, Meulle A, Le Breton A, Dessy C, et al (2012) Regulation of monocarboxylate transporter MCT1 expression by p53 mediates inward and outward lactate fluxes in tumors. Cancer Res 72: 939–948. 22184616 10.1158/0008-5472.CAN-11-2474

[20] Nougayrède JP, Taieb F, De Rycke J, Oswald E (2005) Cyclomodulins: bacterial effectors that modulate the eukaryotic cell cycle. Trends Microbiol 13: 103–110. 15737728 10.1016/j.tim.2005.01.002

[21] Oswald E, Nougayrède JP, Taieb F, Sugai M (2005) Bacterial toxins that modulate host cell-cycle progression. Curr Opin Microbiol 8: 83–91. 15694861 10.1016/j.mib.2004.12.011

[22] Gray MC, Hewlett EL (2011) Cell cycle arrest induced by the bacterial adenylate cyclase toxins from Bacillus anthracis and Bordetella pertussis. Cell Microbiol 13: 123–134. 20946259 10.1111/j.1462-5822.2010.01525.x PMC4137770

[23] Morinaga N, Yahiro K, Matsuura G, Moss J, Noda M (2008) Subtilase cytotoxin, produced by Shiga-toxigenic Escherichia coli, transiently inhibits protein synthesis of Vero cells via degradation of BiP and induces cell cycle arrest at G1 by downregulation of cyclin D1. Cell Microbiol 10: 921–929. 18005237 10.1111/j.1462-5822.2007.01094.x PMC3021990

[24] Nougayrède JP, Homburg S, Taieb F, Boury M, Brzuszkiewicz E, et al (2006) Escherichia coli induces DNA double-strand breaks in eukaryotic cells. Science 313: 848–851. 16902142 10.1126/science.1127059

[25] Putze J, Hennequin C, Nougayrède JP, Zhang W, Homburg S, et al (2009) Genetic structure and distribution of the colibactin genomic island among members of the family Enterobacteriaceae. Infect Immun 77: 4696–4703. 19720753 10.1128/IAI.00522-09 PMC2772509

[26] Samba-Louaka A, Taieb F, Nougayrède JP, Oswald E (2009) Cif type III effector protein: a smart hijacker of the host cell cycle. Future Microbiol 4: 867–877. 19722840 10.2217/fmb.09.60

[27] Iwai H, Kim M, Yoshikawa Y, Ashida H, Ogawa M, et al (2007) A bacterial effector targets Mad2L2, an APC inhibitor, to modulate host cell cycling. Cell. 130: 611–623. 10.1016/j.cell.2007.06.043 17719540

[28] Cover TL, Krishna US, Israel DA, Peek RM Jr (2003) Induction of gastric epithelial cell apoptosis by Helicobacter pylori vacuolating cytotoxin. Cancer Res. 63: 951–957. 12615708

[29] Chang YJ, Wu MS, Lin JT, Pestell RG, Blaser MJ, et al (2006) Mechanisms for Helicobacter pylori CagA-induced cyclin D1 expression that affect cell cycle. Cell Microbiol 8: 1740–1752. 16759223 10.1111/j.1462-5822.2006.00743.x

[30] Turpin W, Humblot C, Thomas M, Guyot JP (2010) Lactobacilli as multifaceted probiotics with poorly disclosed molecular mechanisms. Int J Food Microbiol 143: 87–102. 20801536 10.1016/j.ijfoodmicro.2010.07.032

[31] Azcárate-Peril MA, Sikes M, Bruno-Bárcena JM (2011) The intestinal microbiota, gastrointestinal environment and colorectal cancer: a putative role for probiotics in prevention of colorectal cancer? Am J Physiol Gastrointest Liver Physiol 301: G401–G424. 21700901 10.1152/ajpgi.00110.2011 PMC3774253

[32] Hornef MW, Normark BH, Vandewalle A, Normark S (2003) Intracellular recognition of lipopolysaccharide by toll-like receptor 4 in intestinal epithelial cells. J Exp Med 198: 1225–1235. 14568981 10.1084/jem.20022194 PMC2194240

[33] Chu S, Montrose MH (1995) Extracellular pH regulation in microdomains of colonic crypts: effects of short-chain fatty acids. Proc Natl Acad Sci U S A 92: 3303–3307. 7724557 10.1073/pnas.92.8.3303 PMC42154

[34] Samuel BS, Shaito A, Motoike T, Rey FE, Backhed F, et al (2008) Effects of the gut microbiota on host adiposity are modulated by the short-chain fatty-acid binding G protein-coupled receptor, Gpr41. Proc Natl Acad Sci U S A 105: 16767–16772. 18931303 10.1073/pnas.0808567105 PMC2569967

[35] Sengupta S, Muir JG, Gibson PR (2006) Does butyrate protect from colorectal cancer? J Gastroenterol Hepatol 21: 209–218. 16460475 10.1111/j.1440-1746.2006.04213.x

[36] Bergman EN (1990) Energy contributions of volatile fatty acids from the gastrointestinal tract in various species. Physiol Rev 70: 567–590. 2181501 10.1152/physrev.1990.70.2.567

[37] McNeil NI (1984) The contribution of the large intestine to energy supplies in man. Am J Clin Nutr 39: 338–342. 6320630 10.1093/ajcn/39.2.338

[38] Waldecker M, Kautenburger T, Daumann H, Busch C, Schrenk D (2008) Inhibition of histone-deacetylase activity by short-chain fatty acids and some polyphenol metabolites formed in the colon. J Nutr Biochem 19: 587–593. 18061431 10.1016/j.jnutbio.2007.08.002

[39] Bordonaro M, Lazarova DL, Sartorelli AC (2008) Butyrate and Wnt signaling: a possible solution to the puzzle of dietary fiber and colon cancer risk? Cell Cycle 7: 1178–1183. 18418037 10.4161/cc.7.9.5818

[40] Fukuda S, Toh H, Hase K, Oshima K, Nakanishi Y, et al (2011) Bifidobacteria can protect from enteropathogenic infection through production of acetate. Nature 469: 543–547. 21270894 10.1038/nature09646

[41] Brown D, Bouley R, Paunescu TG, Breton S, Lu HA (2012) New insights into the dynamic regulation of water and acid/base balance by renal epithelial cells. Am J Physiol Cell Physiol In press. 10.1152/ajpcell.00085.2012 PMC3362000 22460710

[42] Lupton JR, Kurtz PP (1993) Relationship of colonic luminal short-chain fatty acids and pH to in vivo cell proliferation in rats. J Nutr 123: 1522–1530. 8395594 10.1093/jn/123.9.1522

[43] Hidalgo IJ, Raub TJ, Borchardt RT (1989) Characterization of the human colon carcinoma cell line (Caco-2) as a model system for intestinal epithelial permeability. Gastroenterology 96(3): 736–749. 2914637

[44] Matsumoto K, Takada T, Shimizu K, Moriyama K, Kawakami K, et al (2006) Effects of a probiotic fermented milk beverage containing Lactobacillus casei strain Shirota on defecation frequency, intestinal microbiota, and the intestinal environment of healthy individuals with soft stools. J Biosci Bioeng 110: 547–552. 10.1016/j.jbiosc.2010.05.016 20580604

[45] Irizarry RA, Hobbs B, Collin F, Beazer-Barclay YD, Antonellis KJ, et al (2003) Exploration, normalization, and summaries of high density oligonucleotide array probe level data. Biostatistics 4: 249–264. 12925520 10.1093/biostatistics/4.2.249

[46] Li C, Wong WH (2001) Model-based analysis of oligonucleotide arrays: expression index computation and outlier detection. Proc Natl Acad Sci U S A 98: 31–36. 11134512 10.1073/pnas.011404098 PMC14539

[47] Schmittgen TD, Livak KJ (2008) Analyzing real-time PCR data by the comparative C(T) method. Nat Protoc 3: 1101–1108. 18546601 10.1038/nprot.2008.73

[48] Laemmli UK (1970) Cleavage of structural proteins during the assembly of the head of bacteriophage T4. *Nature* 227: 680–685. 5432063 10.1038/227680a0

