# Supplementary material for: Epithelial Cell Proliferation Arrest Induced by Lactate and Acetate from Lactobacillus casei and Bifidobacterium breve
Source: PLoS One. 2013 Apr 30;8(4):e63053. doi: 10.1371/journal.pone.0063053 (PMC3639975; doi:10.1371/journal.pone.0063053)
Supplement: Table S3 — Primers used for qRT-PCR in this study. (DOCX) [file pone.0063053.s003.docx]

|  | |  |  |
| --- | --- | --- | --- |
|  |  |  |  |
| Gene | Forward primer (5' -> 3') | Reverse primer (5' -> 3') | Length |
| Cyclin D1 | GATTTGGTCTGCTTGACTTTCC | CAAGAATGTGCCAGAATCAAAC | 106 |
| Cyclin E1 | GAGAGCAGTTCTTCTGGATTGG | GATGCCATGTAACGATCAAAGA | 105 |
| p18^INK4c^(Cdkn2c) | TGCAGGTTATGAAACTTGGAAA | TGAATGACAGCAAAACCAGTTC | 100 |
| p19^INK4d^(Cdkn2d) | TCACACCTGTCCATTGAAGAAG | GGGGTGAGAAAAACAAATGAGA | 111 |
| p21^cip1^(Cdkn1a) | ATGGCTGATCCTTTCTCAGTGT | GGCTAAGGGTAGACAGTCCAGA | 119 |
| p27^Kip2^(Cdkn1b) | ACATATCGCTGACTCCATTGAA | TGCTTTTAGAGGCAGATGGTTT | 106 |
| p53 | ACAATGAAATCTCACCCTACCC | CTTTGCAGAATGGAAGGAAAGT | 100 |
| p57^Kip2^(Cdkn1c) | CTCTAGGGGAATGGTTGTTGAG | TGGTCACCAAGTTTAAGAGCAA | 101 |
| Tob1 | GCCAACCAAGCACAAAGTTATT | AATTCAGCCATGTCCTTGCTAT | 105 |
| GATA-2 | TGGAGAAAGGAGTAGGCAAGAA | ACCCCAAGAACACAAATAGCAC | 100 |
| Ep300 | TCTCAAAAATGCTGGGGATAAG | ACAGTGCTTAGGCTAGGAGTGG | 120 |
| pIgR | CTCTTGGCCTACGATCTGTCTT | ACACCACCATGCTCCTTTAACT | 103 |
| Akp3 | TGGCTCTGTCCAAGACATACAG | CCGATGGTCTTGTAGTTGGTCT | 103 |
| Mct1 | AAAGTGGCGAGCTGCGACGT | CCGGAGTATGCGTGACGCGG | 105 |
| GAPDH | CTTATCAGGCCAAGTATGATG | CAACCTGGTCCTCAGTGTAGC | 100 |
